# Behavioral vaccination policies and game-environment feedback in epidemic dynamics

**DOI:** 10.1038/s41598-023-41420-x

**Published:** 2023-09-04

**Authors:** K. M. Ariful Kabir

**Affiliations:** Department of Mathematics, Bangladesh University of Engineering and Technology, Dhaka, 1000 Bangladesh

**Keywords:** Computational biology and bioinformatics, Evolution, Environmental social sciences, Diseases, Mathematics and computing

## Abstract

Many policymakers have adopted voluntary vaccination policies to alleviate the consequences of contagious diseases. Such policies have several well-established feathers, i.e. they are seasonal, depending on an individual’s decision, adaptive, and control epidemic activity. Here, we study ideas from behavioral epidemiology embedded with a vaccination game and pairwise two-player two-strategy game to represent the environmental feedback in an SVIR model by using a composite information index including disease incidence, vaccine factors and cooperative behavior on a global time scale (repeated season). In its turn, the information index’s game dynamics to participate in the vaccine program (cooperation) is supposed to reflect the feedback-evolving dynamics of competitive cognitions and the environment. The assuming model is described by two different evolutionary game systems connected by an unknown external public opinion environment feedback. The embedded model is described by an inherited system showing a behavioral aspect, i.e. pairwise game indicates an individual’s cooperative behavior, and a vaccine game refers to vaccine-cost influence. This is a novel attempt to stabilize the two different decision processes to pool them into a single index. Extensive simulations suggest a rich spectrum of achievable results, including epidemic control, human behavior, social dilemma, and policy suggestions.

## Introduction

Human’s competitive cognition aspect has provoked great concern. It has been widely studied in recent years due to the rapid development of technology-based information systems, large-scale social networks, blogs, and media^1–3^. On the one hand, social interaction can promote social learning that influences human behaviors; on the other hand, such interactions can reveal individuals at risk of infection. While earlier studies mainly focus on disease patterns, intervention strategies, or internal interactions among individuals^4–9^, the impacts of the external public opinion environment feedback remain unknown. In this work, we study the evolution of human cooperative behavior, vaccine attitude, and environmental feedback in the presence of both beneficial and costly contagions that jointly spread in society.

A substantial theoretical literature has addressed the social dilemma situation for competitive and cooperative human traits that significantly affect epidemic aspects^10^. Still, the cost-dependent vaccination game^11–15^ has received minimal attention. It has been anticipated that individuals may alter their strategies to avoid infection by following an intervention game; however, few models exist that study the optimal control or simple epidemic models. The epidemic disease models includes intervention game model, in which individuals alter their action to seek better intervention during epidemic to avoid diseases^16–18^. A pioneer work studied by Bauch^19^ provides a game-theoretic vaccination model for avoiding disease in an epidemic model. Other researchers have modeled a social learning process that considered vaccination, quarantine, social distancing, and mask-wearing strategies for various updating techniques^20–25^. These works focus on strategic behavior changes that occur on the same time scale (single season) as the disease outbreak. However, we are curious about the long-term epidemic scenario called repeated season to show the evolution of social behavior itself. Regarding the repeated season model, Kuga et al.^11,15^, Kabir et al.^26,27^, Alam et al.^28^, and Arefin et al.^29^ explored the intervention game model for various strategies, including vaccination, treatment, metapopulation, awareness, quarantine, and a two-strain model. In recent years, there has been a surge in research exploring the dynamics of epidemic vaccination, specifically within the context of the vaccination game, both theoretically and through numerical investigations. Kuga et al.^11^ and Kabir et al.^14,26^ introduced SIR/V and SIR/V-UA epidemic models, explored especially pertinent to diseases such as seasonal influenza, as it mirrors specific social contexts and real-world dynamics. These models assumed disease spread over a single season, termed “local time scale,” with strategy updates occurring at each season’s end on a “global time scale,” known as a generation. On the other hand, Bauch et al.^30^ took a different approach by considering scenarios where disease spread and changes in behavior due to social learning happen concurrently within a single season. In our work, we adopt the concept of a global time scale, in contrast to the approach where disease spread, and strategy updates occur on localized scales. A common theme across these systems is the tradeoff between the benefits of social behavior in the context of intervention and disease cost. Most relevant to the proposed work is a model proposed by Kabir et al.^10^, who investigated the evolution of social behavior and dilemma situations in the presence of a pairwise (two by two) game that presented human cooperative behavior on epidemic spreading for the repeated season.

Human cooperative behavior is affected by the payoff of a pairwise game in which two-player and two-strategy interactions are considered for two preferences -cooperation and defection^31,32^. Based on cooperative and defective preferences, a player gets a reward (R) if both players cooperate, and a punishment (P) will receive if both defects. Also, sucker (S) and temptation (T) are the gain payoff for cooperation by one player and defection by the other. Depending on the rescaled properties in terms of universal dilemma strength (SD) game can be presented by the parameters ${D}_{g}^{\prime}$ and ${D}_{r}^{\prime}$ called gamble-intending dilemma and risk-averting dilemma, respectively^33–35^. Based on the magnitude of ${D}_{g}^{\prime}$ and ${D}_{r}^{\prime}$, the game class can be divided into four classes: the Trivial (${D}_{g}^{\prime}<0$ & ${D}_{r}^{\prime}<0$) with no dilemma (), the Prisoner’s Dilemma (${D}_{g}^{\prime}>0$ & ${D}_{r}^{\prime}>0$), Chicken (${D}_{g}^{\prime}>0$ & ${D}_{r}^{\prime}<0$) and Stag Hunt (${D}_{r}^{\prime}>0$, and ${D}_{g}^{\prime}<0$). Most work based on pairwise game theory focuses on dilemma strength (DS). Besides, we are interested in the social dilemma situation known as “social efficiency deficit (SED)” that defined by the payoff difference between social optimum and Nash equilibrium^36,37^.

To realize the diffusion or spreading process and anticipate the evolutionary consequences of competitive cognitions is of great interest for understanding real-world problems^38–40^. Competitive cognition dynamics can imitate the ubiquitous scenarios where individuals are encountered with multiple and mutually exclusive cognitions^41,42^. For example, public health problems, social polarization, pro-vaccination, anti-vaccination, social distancing, mask-wearing, truth, rumors, etc. The conflicting aspect was considered in the infectious disease field to study the co-contagion process extended to describe the competitive diffusion. On the other hand, the state of environment (replete or deplete) can change the preferences of the individuals; as the environment degrades, the cooperation tendency increases^43,44^. Recently, several works have theoretically explored this idea to study the uncertainty of the commons in the resultant eco-evolutionary dynamics that are sported through EGT, describing the strategic relations between the cooperators and the defectors^45–49^. The behavioral aspect present around an individual comprises the environment for the population; hence, based on the above literature review, we use the vaccination game and pairwise game simultaneously. More specifically, this paper is concerned with the game-environment feedback in an epidemic disease model while keeping in mind that the vaccination and pairwise games relate to evolutionary feedback games.

**Figure 1:**
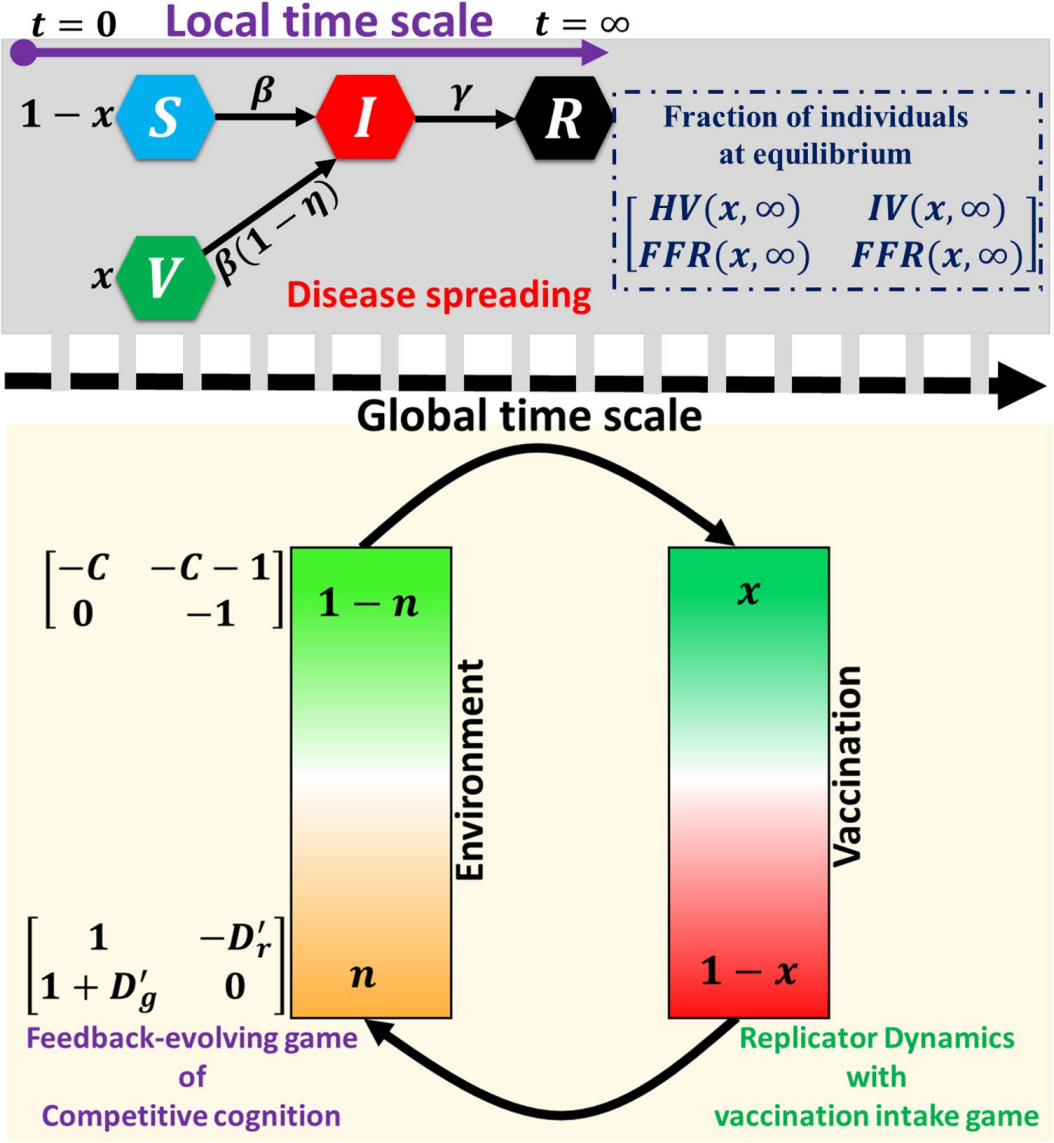
Schematic diagram of the epidemic model in which the population is divided into four states: susceptible (S), vaccinated (V), infected (I), and recovered (R), which applies in the epidemic season on a local time scale. The fraction of cooperators $(x)$ and defectors $(1-x)$ corresponding to the epidemic dynamics changes on the EGT framework in which two game frameworks by shared resource n whose status changes between vaccination game and pairwise game by environmental feedback-evolving game. The evolutionary behavioral game model via environment feedback for vaccination games and the pairwise game is demonstrated on the global time scales with periodic effects.

In this paper, we study the epidemiological SIR/V epidemic model and evolutionary game dynamics of two types of games: vaccination game and pairwise game over the repeated season in which disease spreads on a local time scale and decisions alter on a global time scale (Fig. 1). We first consider an SIR/V epidemic model on a local timescale and study how endemic or disease-free equilibria depends on the population’s social interaction levels. Next, we integrate the evolutionary game theoretical framework to describe the individual’s strategy updating process by the replicator dynamics on a global time scale. Here, cooperation is defined as individuals who participate vaccine program. Finally, we consider another evolution of competitive cognitions game that the replicator dynamics can also characterize in the evolutionary game aspect to how the fraction of individuals will evolve in a public opinion environment. In the result section, we explore the cognition competition between various societal strategies by examining the long-term evolution of social interaction in “Replicator” and “Adaptive” dynamics. We delve deeper into the social dilemma identified in the evolution of social strategies, social change, and behavior-driven epidemiology modeling. Additionally, we incorporate the effects of environmental fluctuation into our epidemic vaccination game model to show their impact on social and behavioral aspects.

Finally, this work introduces a novel model of the Vaccination Game (VG) within a repeated-season framework, simulating an influenza-like epidemic scenario. This model combines the traditional SIR process with evolutionary game theory (EGT) concepts. A key aspect of this VG model is the revised definition of expected payoffs for cooperators (those who agree to vaccination) and defectors (those who don’t commit). Unlike previous VG models that focused solely on vaccination or infection costs, this model incorporates the notion that an individual’s perceived payoff includes rational factors (such as costs) and psychological influences, denoted as the pairwise game (PG). This psychological contribution is captured using a 2-by-2 game framework, scaled by universal dilemma parameters ${D}_{g}^{\prime}$ and ${D}_{r}^{\prime}$. While earlier VG models assumed that the cost of infection accounted for this ‘invisible’ contribution, this study explicitly examines and quantifies this component by introducing PG.

## Model and method

An efficient SIR/V epidemic model is proposed herein to study the influence of voluntary vaccination in which vaccination strategy took place on a global time scale (end of each season). The mean field approximation model explores the imperfect vaccination epidemic dynamics in a well-mixed and infinite population. The fraction of individuals is divided into susceptible (S), infected (I), recovered (R) and vaccinated (V) compartments in which $\eta$ is defined as a constant parameter called vaccine efficacy. The set of differential equations under the imperfect vaccination policy introduced by the efficacy model is^8^,

1 $$\dot{S}(x,t)=-\beta S(x,t)I(x,t)$$

2 $$\dot{V}\left(x,t\right)=-\beta \left(1-\eta \right)V\left(x,t\right)I(x,t)$$

3 $$\dot{I}\left(x,t\right)=\beta S\left(x,t\right)I\left(x,t\right)+\beta \left(1-\eta \right)V\left(x,t\right)I(x,t)-\gamma I(x,t)$$

4 $$\dot{R}(x,t)=\gamma I(x,t)$$

Here, β indicate the disease transmission rate of infection [per day per person] in which the susceptible or vaccinated people become infected. The infected people recovered at the recovery rate γ [per day]. The reproduction number of the systems can be obtained by following inequality condition as follows,

$$\dot{I}=\beta I(x,t)\left[S\left(x,t\right)+\left(1-\eta \right)V\left(x,t\right)\right]-\gamma I(x,t)$$

(i) The derivative $\dot{I}<0,$ if $\beta S(x,t)+\beta V(x,t)\left(1-\eta \right)<\gamma$

(ii) The derivative $\dot{I}>0,$ if $\beta S(x,t)+\beta V(x,t)\left(1-\eta \right)>\gamma$

Thus, the effective reproduction number,

5 $${R}_{e}=\frac{\beta S(x,t)+\beta V(x,t)\left(1-\eta \right)}{\gamma }$$

Here, the existence of stability of the system can be decided by the following proposition. (i) If ${R}_{e}>1$, then the disease-free equilibrium (DFE) is unstable and (ii) for ${R}_{e}<1$, the DFE is stable. Initially, when $t=0$, susceptible and vaccinated individuals are presumed as $V(x,0)=x$ and $S(x,0)=1-x$, respectively, where $x$ indicates the fraction of vaccinators who participate in the voluntary vaccine program (fraction of vaccinators at a certain season). Numerical solutions for the equations are obtained through the explicit finite difference method, and these results will be detailed and discussed in the subsequent section. The two-stage process significantly influences the outcome: equilibrium calculations for the SIR/V time series occur within a single season, while strategy adaptation occurs at each season’s conclusion.

According to the presumed setup described above, four possible fractions of individuals can be obtained at the equilibrium point (end of a season) (Table 1). Four possible fractions are (i) vaccinated and healthy individuals (HV), (ii) vaccinated but infected individuals (IV) (iii) free riding but healthy (SFR), and (iv) free-riding and infected (FFR), evaluated as follows:

6 $$HV\left(x,\infty \right)=x\,\mathrm{exp}[-(1-\eta ){R}_{0}R(x,\infty )]$$

7 $$IV\left(x,\infty \right)=x(1-\mathrm{exp}\left[-\left(1-\eta \right){R}_{0}R\left(x,\infty \right)\right])$$

8 $$SFR\left(x,\infty \right)=(1-x)\mathrm{exp}[{R}_{0}R(x,\infty )]$$

9 $$FFR\left(x,\infty \right)=(1-x)(1-\mathrm{exp}\left[-{R}_{0}R\left(x,\infty \right)\right])$$

where,

**Table 1:** Four fractions of individuals.

	Healthy	Infected
Vaccinated	$HV(x,\infty )$	$IV(x,\infty )$
Non-vaccinated	$SFR(\infty )$	$FFR(\infty )$

10 $$R\left(x,\infty \right)=x\left(1-\mathrm{exp}\left[-\left(1-\eta \right){R}_{0}R\left(x,\infty \right)\right]\right)+(1-x)(1-\mathrm{exp}\left[-{R}_{0}R\left(x,\infty \right)\right])$$

### Payoff structure

Here, we introduce two symmetric games in the context of pairwise (two-player two -strategies) and vaccination games (vaccine cost) to motivate co-evolutionary game theory formalism to characterize the competition of two cognitions. Consider the first symmetric two-player two-strategy game with dilemma strength ${D}_{g}^{\prime}$ and ${D}_{r}^{\prime}$. Competitive games like the donor-recipient and war of attrition models encapsulate pure competition, devoid of social elements. Competitors face four potential strategic interaction options when social influences come into play. Cooperative or mutualistic relationships portray a scenario where both “donor” and “recipient” gain from the collaboration, indicating a situation where both stand to benefit through specific strategies (${D}_{g}^{\prime}<0$ & ${D}_{r}^{\prime}<0$). Altruistic relationships involve the donor incurring a cost to provide the recipient with benefits (${D}_{g}^{\prime}>0$ & ${D}_{r}^{\prime}<0$). Typically, the recipient is kin-related, and donations are unidirectional. Instances where benefits flow reciprocally at a cost might be termed “altruistic,” but close analysis reveals this as stemming from optimized “selfish” strategies. Spite can be seen as the inverse of cooperation, wherein neither party reaps tangible rewards (${D}_{g}^{\prime}<0$ & ${D}_{r}^{\prime}>0$). In most cases, allies are kin-related, and the benefit lies in a less competitive environment for the ally. Selfishness forms the foundational criterion for all strategic choices in game theory – strategies not oriented towards self-preservation and replication tend not to endure (${D}_{g}^{\prime}>0$ & ${D}_{r}^{\prime}>0$).

The second symmetric game contains a vaccination game structure with vaccination and infection cost, $C$ and ${C}_{i}=1$, respectively. The payoff matrix for two games in which the payoffs can be written as follows:

11 $${P}_{G}=\left(\begin{array}{cc}1& -{D}_{r}^{\prime}\\ 1+{D}_{g}^{\prime}& 0\end{array}\right)$$

12 $${P}_{V}=\left(\begin{array}{cc}-C& -C-1\\ 0& -1\end{array}\right)$$

When subjected to two cognitions, an individual prefers either to support the game cognition (cooperative behavior) or vaccine cognition (vaccine behavior). The player gets a payoff or loss during the strategy updating process, which is determined by the supportive level for the two cognitions from the external environment defined as the public opinion environment $n$, $n \in [0, 1]$ (Fig. 1). The environment-dependent payoff matrix of the feedback-evolving game can be written as,

13 $$P=n{P}_{G}+(1-n){P}_{V}$$

14 $$P=n\left(\begin{array}{cc}1& -{D}_{r}^{\prime}\\ 1+{D}_{g}^{\prime}& 0\end{array}\right)+(1-n)\left(\begin{array}{cc}-C& -C-1\\ 0& -1\end{array}\right)$$

where $n$ and $(1 - n)$ imply the environments for anticipating the game cognition and the vaccine cognition, respectively.

The expected payoff (fitness) of cooperators (vaccinated) and defectors (non-vaccinated), ${\pi }^{C}$ and ${\pi }^{D}$, are,

15 $${\pi }^{C}=HV\left(x,\infty \right)\left[n-C\left(1-n\right)\right]+IV\left(x,\infty \right)[\left(-{D}_{r}^{\prime}\right)n+\left(1-n\right)\left(-1-C\right)]$$

16 $${\pi }^{D}=SFR\left(x,\infty \right)\left(1+{D}_{g}^{\prime}\right)n-FFR\left(x,\infty \right)(1-n)$$

Let us now consider replicator equation for pairwise Fermi rule known as nonlinear dynamics. The transition probability for pairwise nonlinear replicator dynamics is given by,

17 $${P}_{{\pi }^{D}\leftarrow {\pi }^{C}}=\frac{1}{1+exp[-({\pi }^{C}-{\pi }^{D})/\kappa ]}$$

18 $${P}_{{\pi }^{C}\leftarrow {\pi }^{D}}=\frac{1}{1+exp[-({\pi }^{D}-{\pi }^{C})/\kappa ]}$$

Here, ${P}_{{\pi }^{D}\leftarrow {\pi }^{C}}$ and ${P}_{{\pi }^{C}\leftarrow {\pi }^{D}}$ present the pairwise benefit function, we can then illustrate the non-linear replicator equations as,

19 $$\dot{x}=x\left(1-x\right)[{P}_{{\pi }^{D}\leftarrow {\pi }^{C}}-{P}_{{\pi }^{C}\leftarrow {\pi }^{D}}]$$

Finally, to quantify environmental evolution dynamics $n$, the ratio of enhancement rates to degradation rates $\theta >0$ is introduced. Here, only the resource enhancement by the environment/ agent is considered to alter the dynamics. In this aspect, the cooperators help to enhance, while the defectors act to diminish. The environmental evolution dynamic of the shared resource is written by,

20 $$\dot{n}=\epsilon n\left(1-n\right)[-1+(1+\theta )x]$$

In which $\epsilon$ is the relative feedback rate of the environment to cognition dynamics. The logistic term $n(1 - n)$ assures $n \in [0, 1]$.

The outcome is significantly shaped by a two-stage process: first, equilibrium calculations for the SIR/V time series occur within a single season, and second, strategy adaptation occurs after each season. Within our epidemic disease model, specifically in Eqs. (1–4), we introduce a second variable, “$x$,” alongside the time variable denoted as “$t$.” This “$t$” signifies changes occurring at a local time step, a term we use in our terminology. Importantly, equilibrium values for all compartments are reached as “$t$” approaches at equilibrium ($t-\infty$). Using these equilibrium values, the strategy updating process, guided by Eq. (19), is executed, leading to updated “$x$” values for vaccination (and, equivalently, “$1-x$” for susceptibility). Following this, disease dynamics unfold over a time scale marked as “$t$,” progressing cyclically within a global time scale known as a generation. This iterative progression ultimately yields the model’s outcomes.

## Result and discussion

The model established in this study considers the epidemic vaccination dynamics and evolutionary game theory approach to the infinite and well-mixed population with dispersed disease transmission, vaccine cost, vaccine efficacy, pairwise cooperative game, and game environment feedback. We have examined the impact of vaccine hesitancy, acceptance and homophily, i.e., assortative merging by vaccination cost, game dilemma strength and feedback degradation rate on the disease final epidemic size and vaccination coverage. The preference for individuals to associate and interact with others, known as vaccination hesitancy or homophily, can arise in human society due to various reasons such as personal belief, vaccine reliability, cooperative attitude, vaccine cost, geography, and environmental factors. Here, we developed a workflow to explore human behavior’s impact on controlling an epidemic in a global time step from the perspective of environmental feedback.

### Pairwise game cognition versus vaccination game cognition

**Figure 2:**
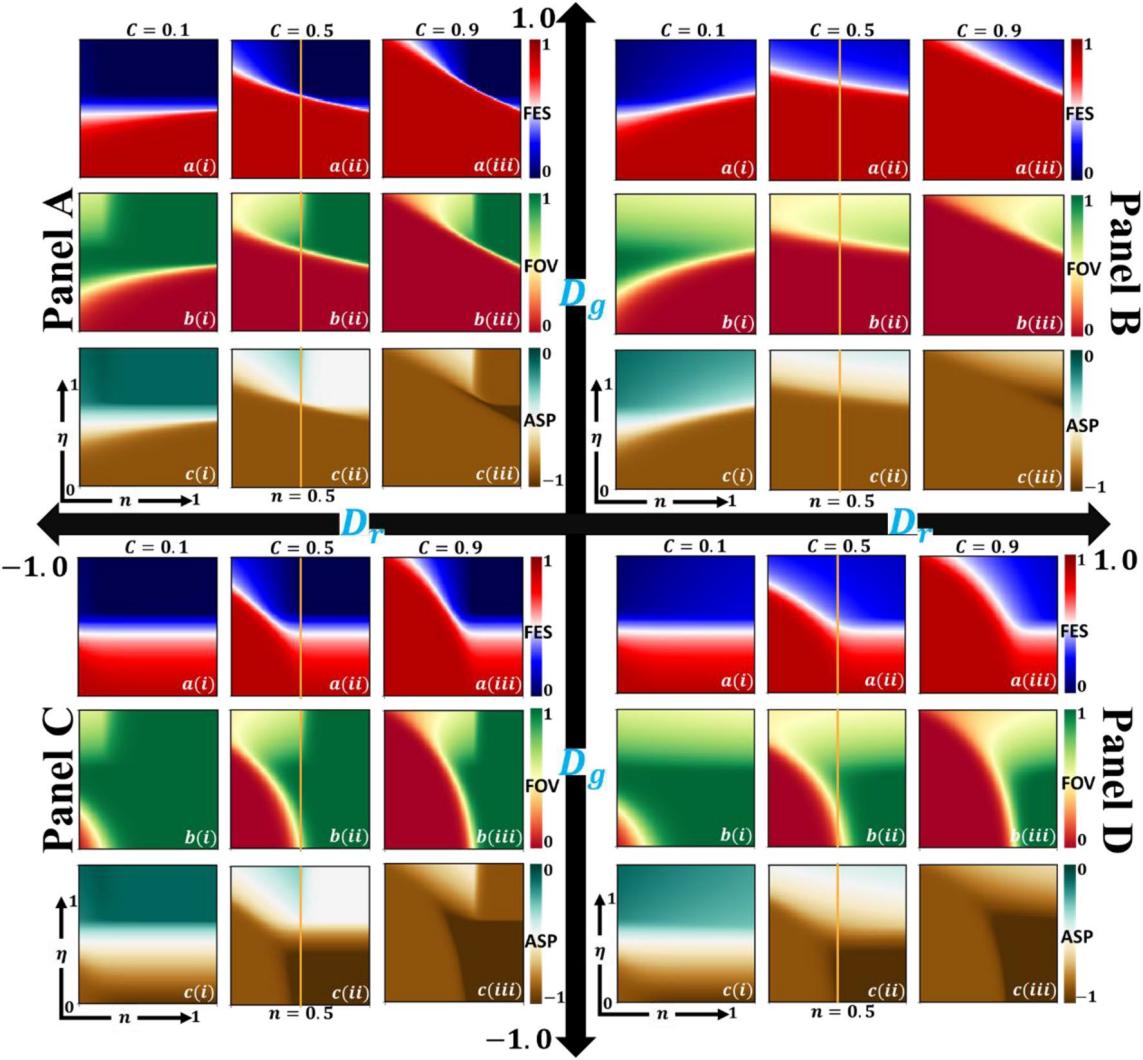
Example heatmaps of (a-*) final epidemic size (FES), (b-*) fraction of vaccinators (FOV), and (c-*) average social payoff (ASP) for four-game classes: Panel A (Chicken), Panel B (Prisoner’s dilemma), Panel C (Trivial), and Panel D (Stag hunt). The 2D phase portrait depicted along vaccine efficiency ($\eta$) and environmental factor ($n$) for various costs (*-i) C = 0.1, (*-ii) C = 0.5, and (*-iii) C = 0.9. Related parameters (Table 2) are, $\beta =0.83333, \gamma =0.3333,$ and $\kappa =0.1$^8^.

This subsection performs under the condition of varying the environmental factor $n$ at a constant rate that the environmental factor has no evolution dynamics; to explore the influence of the factor $n$ on the epidemic and EGT. To focus on the cognition of pairwise vs vaccination game along with vaccine efficacy and factor n in a steady state position, Fig. 2 presents several diagrams by considering the four-game classes: Prisoner’s dilemma, Trivial, Chicken, and Stag Hunt for vaccine cost $C=0.1$, $0.5$ and $0.9$. Here, the fraction of the population under EGT is displayed by the graph of (a-*) final epidemic size (FES), (b-*) vaccination coverage and (c-*) average social payoff, respectively. Meanwhile, sub-panel (*-i), sub-panel (*-ii) and sub-panel (*-iii) display for varying vaccination cost of $C=0.1$, $C=0.5$ and $C=0.9$.

**Table 2:** Parameters used in this work, their respective definitions, numerical values, and corresponding sources.

Symbols	Meanings	Value	References
$\beta$	The disease transmission rate	0.8333	^4,11,14^
$\gamma$	Recovery rate	0.3333	^4,11,14^
$\eta$	vaccine efficacy	Varied [0,1]	^11,14,15^
$x$	Fraction of vaccinator	Varied [0,1]	^11,14,15^
${C}_{v}$	Vaccination cost	Varied [0,1]	^11,14,15^
${C}_{i}$	Infection cost	1.0	^11,14,15^
${C}_{r}$	Relative vaccination cost	Varied [0,1]	^11,14,15^
$\kappa$	Sensitivity of individuals	0.1	^11,14,15^
${D}_{g}^{\prime}$	Gamble-intending dilemma	Varied [− 1,1]	^15,34^
${D}_{r}^{\prime}$	Risk-averting dilemma	Varied [− 1,1]	^15,34^

The case of the Prisoner’s dilemma (AllD) is shown in Panel B, where dilemma strength parameters are ${D}_{g}^{\prime}>0$ and ${D}_{r}^{\prime}>0$. The varying propensity of vaccine efficacy from low to high shows the significant reduction of final epidemic size (red to blue) and increase in vaccination coverage (red to green). Let us be concerned about the environmental factor $n$ on the area with $n<0.5$ in case of vaccination game priority, which implies that the highest VC occurred for lower vaccine cost $(C=0.1)$. However, higher vaccine cost illustrates a lower possibility of vaccine coverage. This monotonic declining tendency happens because of n values. Human is more prone to depend on a prisoner’s dilemma-based cooperative game when n is higher $(n>0.5)$. Thus, it illustrates that the vaccination game can lessen the threat of infection when vaccine cost is minimal. This is because the propensity of n inclined to the vaccination game for $n<0.5$ ensures the environmental feedback dynamics for human adaptation strategies, regardless of being leaned on the vaccination game or cooperative game. As a whole, it can be obtained that higher $\eta$ with a higher $n$ increase in the vaccination coverage exhibits a substantial impact, reducing the infection. Meanwhile, comparatively reasonably lower vaccine costs present vaccine higher outcomes.

Now let us concentrate on the outcomes for the Trivial (AllC) game in Panel C that are generated following a similar procedure as explained above. As a prime example, the case illustrates for $n>0.5$ on vaccination coverage (b-*), we observed that most of the individuals are inclined to participate in vaccine programs (green). This comes from the fact of a fully cooperative situation (AllC) from the trivial game in which society reached its highest cooperative positions. However, it is stated that the overall desired vaccination coverage for $n>0.5$ would be relatively higher, which is appeared in sub-panel (b-*). Nevertheless, in this case $(n>0.5)$, the FES (a-*) for lower vaccine efficacy seems higher than higher $\eta$, which indicates the vaccine reliability of imperfectness of vaccination. Another crucially important parameter, $C$, as the cost for vaccination, has been played here to persuade vaccination level to control contagious diseases. The vaccination coverage area (green) is enhanced when vaccine cost is lower and compressed for higher $C$.

By comparing Panel B and Panel C, we can see that the PD and Trivial resent opposite results in which PD exhibits less impact to participate in the vaccine program when $n>0.5$. Conversely, a different propensity was found for $n<0.5$ compared to the two cases, ensuring that vaccination cost displays a substantial impact.

In Panel A, CH demonstrates an overreaching of vaccination uptake then PD for reasonably higher and lower vaccine cost; the AllC region emerged. On the other hand, for individuals with both cooperative and defective minds in the stag hunt game (Panel D), yet with less vaccine effectiveness, it is always better for a society to participate in a vaccination program.

Another interesting phenomenon is examined for all four cases, leading to individuals being protected themselves by herd immunity to get the benefit of free-riding. In sum, when the vaccine efficacy is relatively higher (upper region of subpanel (b-*)), the beneficial one is vaccinated, yet, unvaccinated also get more benefits.

Lastly, the average social payoff (ASP) is displayed in subpanel (c-*). The lower cost with higher vaccine efficacy improves the payoff for the case of $n<0.5$, and the Trivial game (Panel C) resent a higher payoff when $n>0.5$ that the above discussion can uphold.

### Environment, n versus vaccinators, x

**Figure 3:**
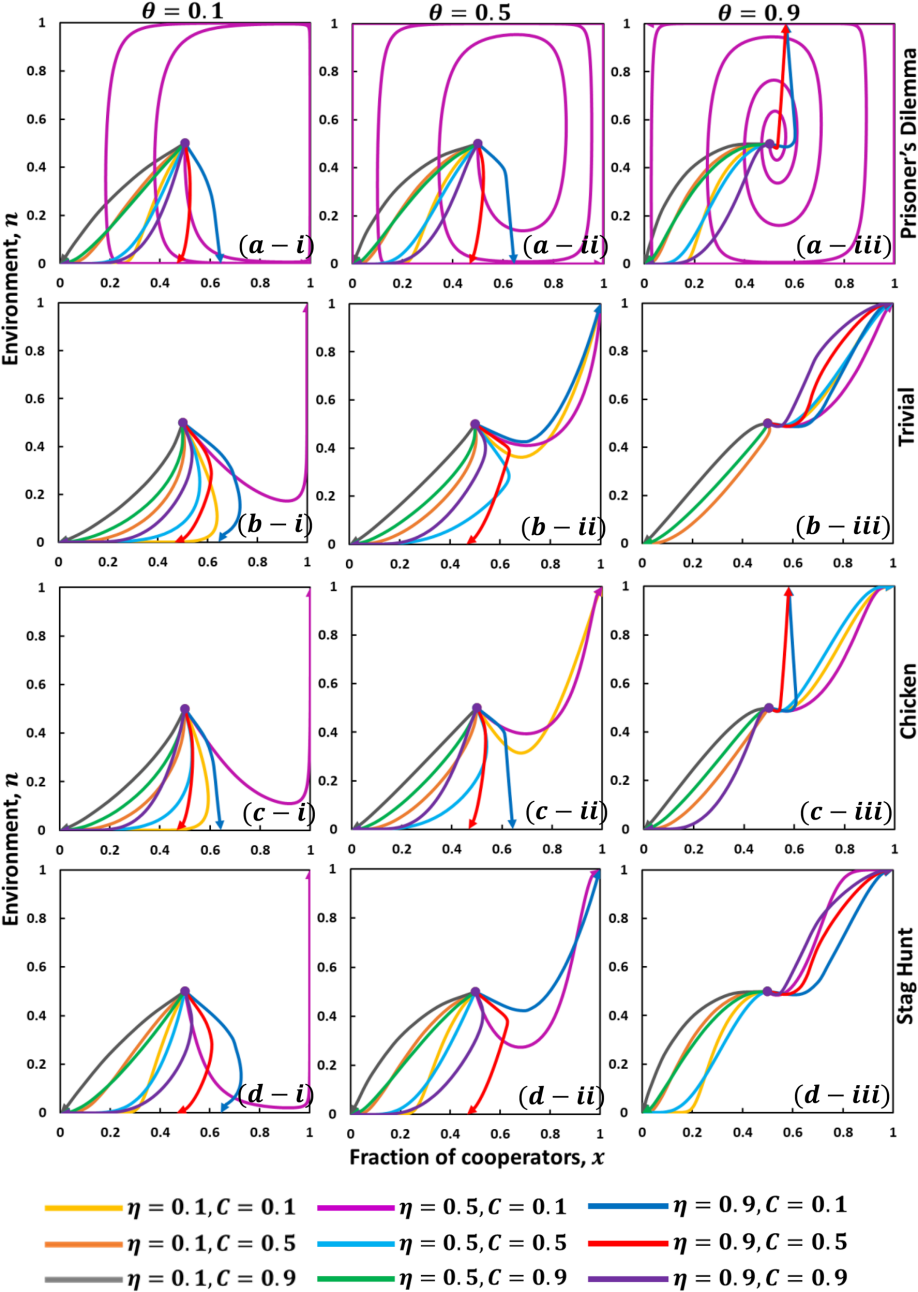
Phase trajectories projected on $x-n$ plane (vaccinators and environment) under the influence of evolutionary game theory on global time scale. The case of (a-*), (b-*), (c-*) and (d-*) for Prisoner’s Dilemma, Trivial, Chicken and Stag Hunt game with $\theta =0.1, 0.5,$ and $0.9$ are considered here. The figure present how an initial condition $({x}_{0},{n}_{0})=(0.5, 0.5)$, approaches either ${n}^{*}=0$ or ${n}^{*}=1$ varying parameters of $\eta$ and $C$. The solid yellow, orange, gray, magenta, sky-blue, green, blue, red and purple lines mean trajectory path for $(\eta , C)= (0.1, 0.1), (0.1, 0.5), (0.1, 0.9), (0.5, 0.1), (0.5, 0.5), (0.5, 0.9), (0.9, 0.1), (0.9, 0.5)$ and (0.9, 0.9), respectively. Related parameters (Table 2) are, $\beta =0.83333, \gamma =0.3333,$ and $\kappa =0.1$^8^.

For the previous results, we considered only evolutionary games for vaccination behavior that studied whether individuals are taking vaccines or not with constant n. However, it does not assess whether individuals have an environmental-feedback evolution to adopt their respective strategies. To explore, we now consider dynamic time-dependent environmental evolution dynamics on the repeated season. In Fig. 3, each subplot displays the time series of $n$, the time series of $x$, and the phase trajectory projected on the $x-n$ plane on the global time scale. Explicitly, sub-plot (a-*), (b-*), (c-*), and (d-*) demonstrate four types of games, namely, Prisoner’s dilemma, Trivial, Chicken, and Stag Hunt. Also, sub-plot (*-i), (*-ii), and (*-iii) display the values of the ratio of enhancement rates to degradation rates $\theta =0.1, 0.5$ and $0.9$, respectively. Here, the figure presents how an initial setting $(x(0), n(0))=(\mathrm{0.5,0.5})$ approaches either $n(\infty )=0$ or $n(\infty )$=1, with a monotonic change in the values of vaccine efficacy and its associated cost. The solid lines (see figures) indicate various trajectory lines for multiple vaccine efficacy and cost combinations.

In the case of the Prisoner’s dilemma game (a-*), when vaccine efficiency is lower, the cost is higher, and $\theta =0.1$, the initial state is pulled toward a stable point that corresponds to $(x,n)=(\mathrm{0,0})$. But, for higher vaccine efficiency and lower cost, $x$ increases as well (red and blue). None of these trajectories are limit cycles observed for $(\eta , C)= (\mathrm{0.1,0.1})$, $(\mathrm{0.1,0.5})$, $(\mathrm{0.1,0.9})$, $(\mathrm{0.5,0.5})$, $(\mathrm{0.5,0.9})$, and $(\mathrm{0.9,0.9})$. However, the orientation of limit cycle orbits in the phase plane is observed for $(\eta , C)= (\mathrm{0.5,0.1})$ show counterclockwise (Magenta). The insight phenomenon is as follows. When cooperation is preferred $(x=1)$, the system will move from $(x,n)=(\mathrm{0,0})$ to $(x,n)=(\mathrm{1,0})$. Then, as cooperators boost the environment, the system will be approached closer to $(x,n)=(\mathrm{1,1})$. Traitors will occupy an environmental state, and the system will turn to a near $\left(x,n\right)=(\mathrm{0,1})$ state. Finally, in an environment led by traitors, the environment state will be tainted, and the system will be nearer to $(n,x)=(\mathrm{0,0})$. Thus, the intuition of cooperation, defection, attraction, and environmental feedback leads to complete circumvention of the system changes in size as efficacy and cost of vaccination change. The case explanation, as mentioned above, can be analogously understood as the payoff matrix for the Prisoner’s dilemma game.

We further discuss how our standard parameters implicate the position of the equilibrium and the interior saddle point for $\theta$ changes, the relative strength of enhancing the environment by cooperators versus decreasing the environment by traitors, which confines the interest of environmental feedback. Generally, $n$ is only affected by $\theta$; the time scale of $n$ enhances as $\theta$ increases, presenting more cooperators participating in the social improvement if each has a higher impact. For $\theta =0.9$, present that $n$ is increased and approaches its maximum point $(n=1)$ when vaccine efficiency is high, and the cost is low (red and deep blue). These dynamic properties highlight the importance of the interior saddle point for more cooperators participating in the social innovation in how vaccine cost and efficiency impact participation in the vaccine program.

There are four possibilities to consider corresponding to various combinations among the relative values of ${D}_{g}^{\prime}$ and ${D}_{r}^{\prime}$ depicted in Fig. 3; Prisoner’s dilemma game for ${D}_{g}^{\prime}>0$ and ${D}_{r}^{\prime}>0$ (sub-plot a-*), Trivial game for ${D}_{g}^{\prime}<0$ and ${D}_{r}^{\prime}<0$ (sub-plot b-*), Chicken game for ${D}_{g}^{\prime}>0$ and ${D}_{r}^{\prime}<0$ (sub-plot c-*), and Stag hunt game for ${D}_{g}^{\prime}<0$ and ${D}_{r}^{\prime}>0$ (sub-plot d-*). Of these, we have already analyzed the dynamics behavior arising in the first cases when ${D}_{g}^{\prime}>0$ and ${D}_{r}^{\prime}>0$ (PD). The second case corresponds to domination by a cooperating strategy. The system will converge to $(x,n)=(\mathrm{1,1})$ when $\eta =0.5$ and $C=0.1$ for small $\theta$ $(=0.1)$. However, increasing $\theta$ presents several cases to be concurrent to $(x,n)=(\mathrm{1,1})$. Therefore, cooperation will be the dominant strategy for higher values of $\theta$ if the vaccine is reliable and cheap.

Now, comparing the sub-plot (b-*) and (d-*) for TR and SH, the trajectory shows a similar tendency. The chicken game shows a similar movement with TR when $\theta$ is lower and identical to PD when $\theta$ is higher (the vaccine is reliable at a lower cost).

### Inclusion of enhancement rates to degradation rates, $\theta$

**Figure 4:**
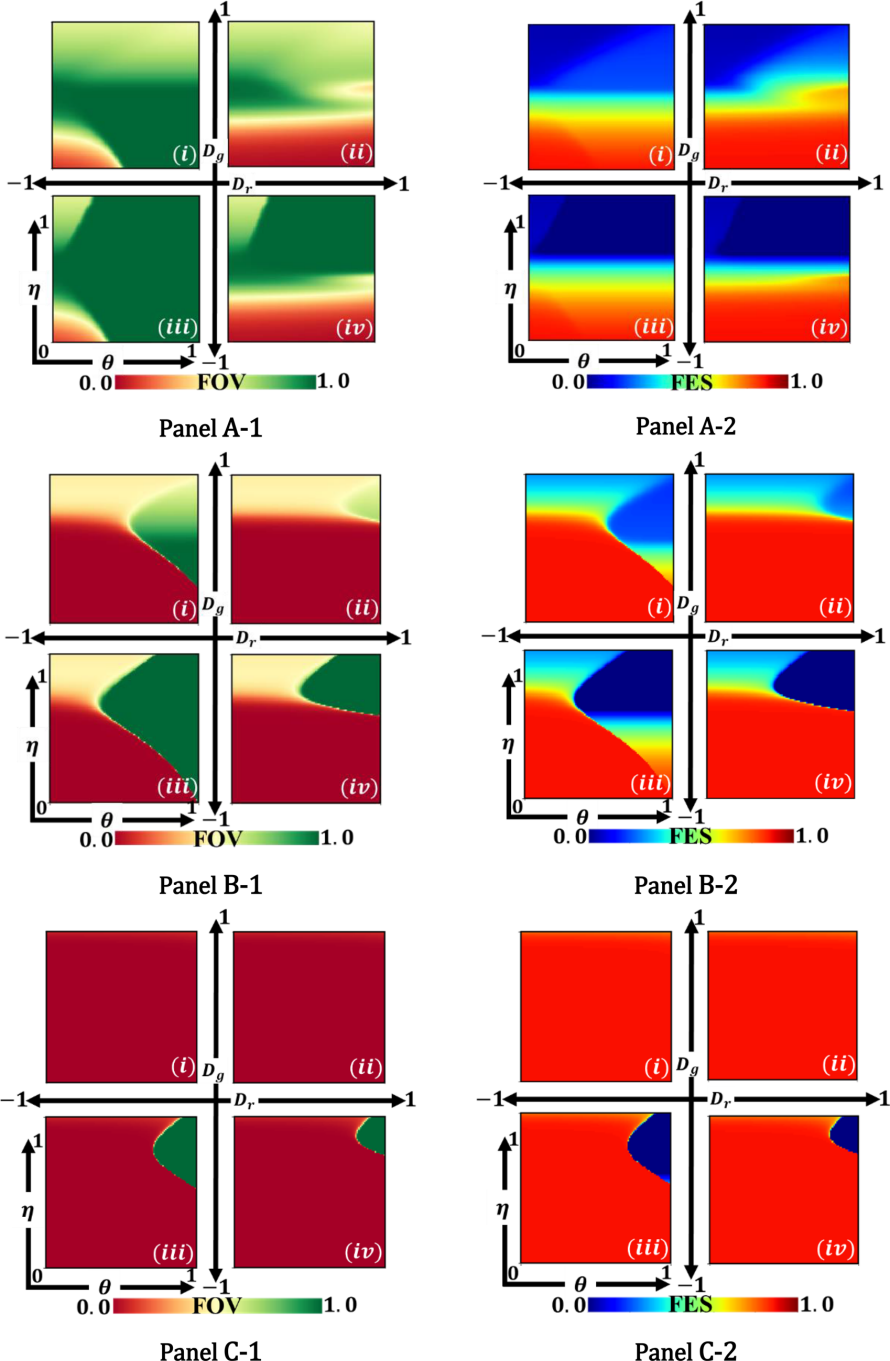
Example heatmaps of (*-1) fraction of vaccinators (FOV), and (*-2) final epidemic size (FES), for four-game classes: (i) Chicken, (ii) Prisoner’s dilemma, (iii)Trivial, and (iv) Stag hunt. The 2D phase portrait depicted along $\eta$ and $\theta$ for different types of costs (A-*) $C=0.1$, (B-*) $C=0.5$, and (C-*) $C=0.9$. Related parameters (Table 2) are, $\beta =0.83333, \gamma =0.3333,$ and $\kappa =0.1$^8^.

The last section explored the idea of evolutionary environmental feedback dynamics and vaccination behaviour under which the strategy of the commons is avoided and accepted for possible settings. In particular, the system converges to an intermediate environment and vaccination coverage when the cumulative intensity of vaccination effectiveness, vaccine cost and $\theta$ exist in favour of individuals who want to participate vaccine program. However, in this section (Fig. 4), we consider the 2D phase diagram of the fraction of vaccinators (FOC) (A-*) and final epidemic size (FES) (B-*) along with vaccine efficiency and $\theta$ for varying vaccine costs that analyze the corresponding factors encompassing four games.

As a holistic tendency, note that a larger FOC and smaller FES are obtained for the lower vaccine cost for $C=0.1$ (Panel A-i). However, A smaller FOC can be observed when vaccine cost is comparatively higher (C = 0.9), which delivers a higher FES region. This is because cheaper vaccine cost ensures more significant participation of vaccine program in controlling diseases. Now, throughout the case along $\eta$, although it is expected that increasing $\eta$, there is no hope for individuals to defect (AllC strategy), we could see some non-vaccinated regions. Regarding lower vaccine cost in Panel A-1 (iii), FOC shows a non-vaccinated area for both lower and higher vaccine efficiency regions. Above critical vaccine effectiveness, the area is divided into two portions; when efficiency is lower, individuals avoid the vaccine, and herd immunity emerges (get free riding) for higher reliability of the vaccine. This implies that individuals refrain from vaccines either for untrustworthy or to get the advantages of free riding. Consequently, in panels B and C, it is found that increasing vaccine costs increase the non-vaccinated area. Therefore, the vaccination is performed better when the efficiency and vaccine cost is under a certain level that the health authority should be concerned about before applying vaccine policy.

In the case of $\theta$, the relative strength of enhancing the environment enhanced the vaccination (cooperation) for increasing the values of $\theta$ in TR, SH, and CH game classes in Panel A. However, the inverse scenario was only observed for higher $\theta$ in PD cases (A-1 (ii)), resulting in a desperate situation with relatively high FES. Finally, by comparing, Panel A for lower vaccine cost presents the most excellent performance to lessen contagious disease compared with the others two Panels. On the other hand, Panel C for higher vaccine cost demonstrates an entirely defective region (no-vaccination) for PD and CH games, meaning society has already attained its wholly unsatisfactory situation (AllD) in which there is no hope for participating vaccine program to control contagious diseases.

### Fraction of individuals expected to adhere to vaccine

**Figure 5:**
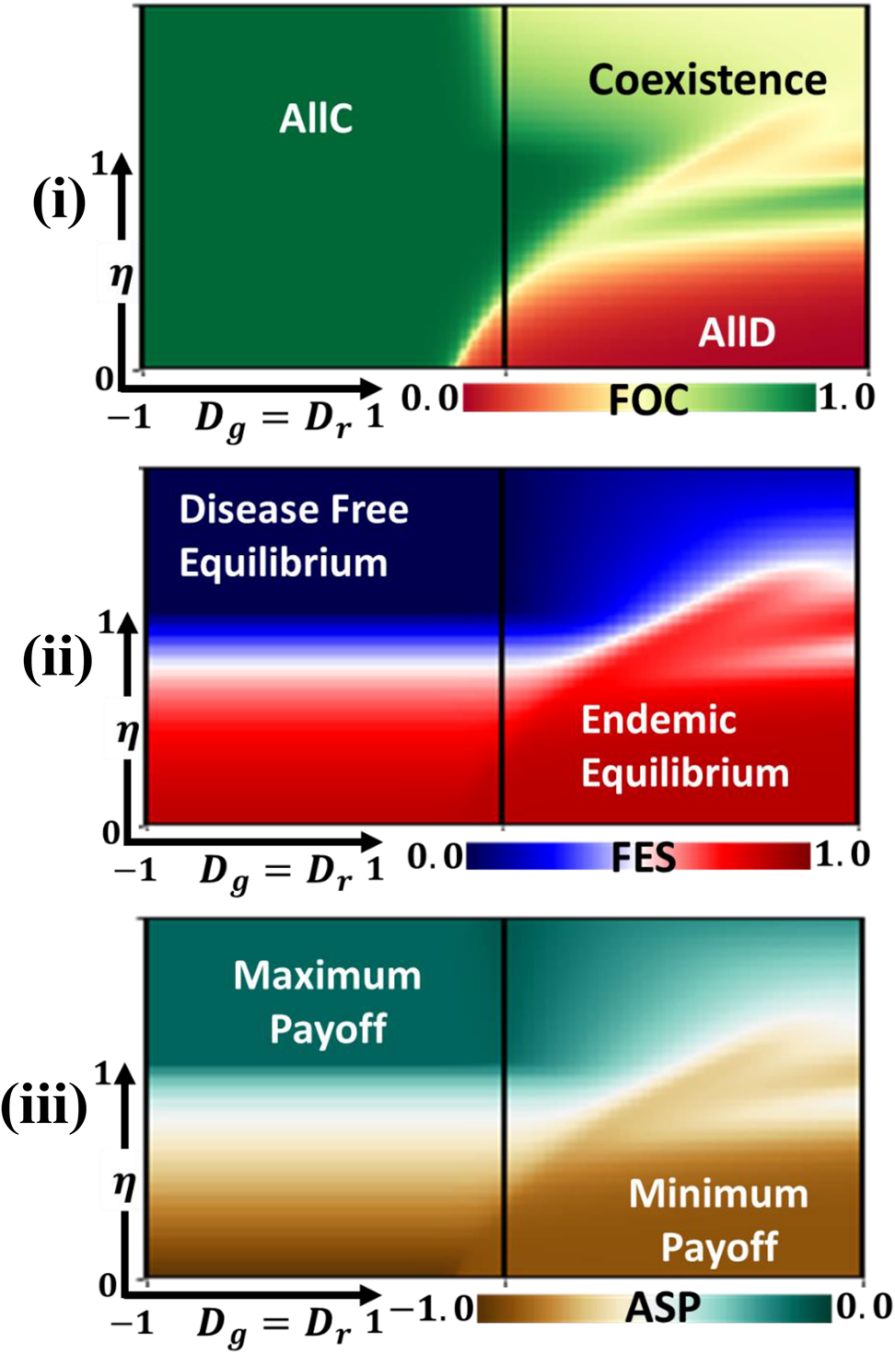
Example heatmaps of (i) fraction of vaccinators (FOV), (ii) final epidemic size (FES), and (iii) average social payoff (ASP) along $\eta$ and ${D}_{g}^{\prime}={D}_{g}^{\prime} [-\mathrm{1,1}]$ Related parameters (Table 2) are, $\beta =0.83333, \gamma =0.3333, \theta =0.9, C=0.1$ and $\kappa =0.1$^8^.

The advantages of vaccination can come in different forms. For example, it can save individuals from disease, reduce the probability of transmission, and generate herd immunity for free riding, as the benefits occur over a longer time scale than other provisions. In Fig. 5, the combined effect of vaccine efficiency and universal dilemma strength have been considered in evaluating the (i) FOC, (ii) FES and (iii) ASP for fixed vaccine cost $C=0.1$ and $\theta =0.9$.

Let us start by thinking of a hypothetical cooperative society (AllC) in which universal dilemmas strength (UDS) are negative ${D}_{g}^{\prime}<0$ and ${D}_{r}^{\prime}<0$. Higher vaccine efficiency and negative UDS increase vaccine expressively, which seems crucial because it makes individuals comply with taking the vaccination. However, we can recognize exceptions phenomena when increasing UDS, which is that almost AllD regions observed for lower $\eta$ and coexistence area are seen for higher $\eta$, respectively. It suggests that individuals are not interested in participating in vaccine programs for unreliable vaccines, and increased vaccine reliability reduces the FOC, indicating the emergence of herd immunity.

Further, for positive dilemma strength (${D}_{g}^{\prime}>0$ and ${D}_{r}^{\prime}>0$), we could see three possible scenarios: AllC, AllD, and coexistence. In this case, lower $\eta$ values present lower FOC that arises AllD region (PD) and lead to a high risk of infection. In the issue of intermediate vaccine effectiveness, the AllC region appeared, still expecting everyone to get vaccinated. Individuals’ choices would lead to no vaccinations, but it would be advantageous if others were immunized. For high vaccine reliability, vaccine uptake becomes very heterogeneous; we observed a coexistence scenario (CH)-such a case is particularly likely if there is increased reliability with misleading information. Similarly, Panel (ii) and Panel (iii) for FES and ASP display a similar overall tendency; vaccine practice would likely be optimal if it reduces the transmission of contagious diseases, despite its societal benefits in risk averting the situation.

## Conclusions

The enduring pandemic has illustrated the significance of human behavior in epidemic response and intervention policies to control and eradicate diseases. Evolutionary game theory effectively studies human behavioral dynamics and the incentive-driven dynamical evolution in natural systems. In the current endeavor, we embedded three dynamical processes in the same framework on EGT: vaccination game, pairwise game, and environmental feedback evolving game. We developed a coevolutionary game theoretical approach that first integrates the feedback loop between the vaccination game and pairwise game that incorporates between environment and EGT on a global time scale under epidemic disease dynamics. In the feedback-evolving game, replicator dynamics was encompassed by assuming that the state-dependent payoff matrix can be expressed as a combination of two separate payoff matrices (vaccination and pairwise). The results demonstrated how various dynamics and factors could influence epidemic results when cooperators dominate in change environments, leading to a shift in incentives that favors defection or cooperation. This study also provides insights into the oscillating dynamics of Prisoner’s dilemma games, driven by the strategy-dependent environmental feedback from vaccine behavior. This has significant implications for several issues, such as the variation of vaccine reliability, vaccination cost, vaccine dilemma, reluctance, refusal, free riding, and disease incidence. Further, results present that unyielding cooperation can emerge if the environmental feedback relative to the strategy is high enough. Regardless of the advantages made in this area, there have been few efforts to apply this embedded theoretical framework to the evolution of cognition, such as beliefs, ideas, behaviors, social influence, convincing, cost, and benefits. Explicitly, it remains unknown how the environment of public conduct and opinion affects the competition among cognitions on an epidemiological aspect, which is a widespread and crucial issue in a society that is becoming increasingly unified.

## Data Availability

The datasets generated and/or analyzed during the current study are not publicly available due to original code but are available from the corresponding author on reasonable request.
